# Restless legs syndrome and ferritin levels in older adults with dementia: a cross-sectional study

**DOI:** 10.1590/1980-5764-DN-2024-0218

**Published:** 2025-04-07

**Authors:** Érica Dayanne Meireles Leite, Madson Alan Maximiano-Barreto, Letícia Lambert, Victor Oliveira Wercelens, Álan Luiz Éckeli, Marcos Hortes Nisihara Chagas

**Affiliations:** 1 Universidade de São Paulo, Faculdade de Medicina de Ribeirão Preto, Departamento de Neurociências e Ciências do Comportamento, Ribeirão Preto SP, Brazil.; 2 Universidade de São Paulo, Grupo de Estudos e Pesquisas em Saúde Mental, Cognição e Envelhecimento, Ribeirão Preto SP, Brazil.; 3 Instituto Bairral de Psiquiatria, Itapira SP, Brazil.; 4 Hospital São Silvestre, Aparecida de Goiânia GO, Brazil.

**Keywords:** Aging, Dementia, Ferritins, Restless Legs Syndrome, Envelhecimento, Demência, Ferritinas, Síndrome das Pernas Inquietas

## Abstract

In cases of dementia or major neurocognitive disorder, restless legs syndrome has not been extensively studied because the diagnosis relies on clinical assessments and self-reports from patients.

**Objective:**

The aim of this study was to investigate the association between Willis-Ekbom disease/restless legs syndrome and iron-deficiency anemia in older adults with dementia.

**Methods:**

A cross-sectional study was conducted with 70 older adults diagnosed with dementia and restless legs syndrome at a psychogeriatric clinic in the state of São Paulo, Brazil. The participants answered data collection instruments addressing sociodemographic characteristics, restless legs syndrome, neuropsychiatric symptoms, sleep quality, daytime sleepiness, and cognitive function. Creatinine, ferritin, red blood cells, hemoglobin, and hematocrit were determined by blood exams (the latter of which was collected from the patient records).

**Results:**

The sample was composed predominantly of individuals with mixed dementia (i.e., Alzheimer's disease+vascular dementia). Women accounted for 55.7% of the sample, with a mean age of 77.80±9.36 years. The prevalence of restless legs syndrome among the participants was found to be 15.7%. Individuals with this syndrome had greater frequencies of neuropsychiatric symptoms, poor sleep quality, higher BMI, and lower ferritin levels (p<0.05).

**Conclusion:**

The prevalence of restless legs syndrome among older adults with dementia was 15.7%, and individuals with this syndrome had ferritin deficiency.

## RESUMO.

Na demência ou transtorno neurocognitivo maior, a síndrome das pernas inquietas é pouco estudada em razão de o diagnóstico ser clínico e depender da entrevista e do autorrelato dos pacientes.

**Objetivo:**

Avaliar a relação entre doença de Willis-Ekbom/síndrome das pernas inquietas e anemia ferropriva em pessoas idosas com demência.

**Métodos:**

Um estudo transversal foi conduzido com 70 pessoas idosas com diagnóstico de demência e síndrome das pernas inquietas em um ambulatório de psicogeriatria de uma cidade do interior de São Paulo, Brasil. As pessoas idosas preencheram instrumentos de caracterização sociodemográfica, medidas para avaliar a síndrome das pernas inquietas, sintomas neuropsiquiátricos, qualidade do sono, sonolência e cognição. Também foram coletados dados sanguíneos — os níveis de creatinina, ferritina, hemácias, hemoglobina e hematócrito, este último coletado nos prontuários dos pacientes.

**Resultados:**

A amostra é composta, na maioria, de pessoas idosas com demência mista (i.e., doença de Alzheimer+demência vascular), com 55,7% dos pacientes do sexo feminino e média de idade de 77,80 anos (±9,36). Este estudo identificou frequência de 15,7% da síndrome das pernas inquietas. Os pacientes com a síndrome apresentam mais frequência de sintomas neuropsiquiátricos, pior qualidade do sono, maior índice de massa corporal e menores níveis de ferritina (p<0,05).

**Conclusão:**

Uma frequência de 15,7% foi identificada da síndrome das pernas inquietas entre pacientes com demência. Além disso, pacientes com a síndrome apresentam deficiência de ferritina.

## INTRODUCTION

Major neurocognitive disorder (MND) (i.e., dementia) is a common condition throughout the world^1^, accompanying the increase in the older population^2^. MND is characterized by a significant decline in one of the six neurocognitive functions (i.e., executive function, complex attention, language, learning and memory, perceptive-motor skills, and social cognition)^3^. Alzheimer's disease is the most common form of dementia among older adults^4^. Other types include dementia related to Parkinson's disease, vascular dementia, frontotemporal dementia, and Lewy body dementia^5^.

Besides cognitive impairment, non-cognitive signs are also seen in individuals with dementia, such as psychotic disorders^6^, anxiety^7^, depression^8^, and the like. Signs may even emerge in the preclinical phase of dementia and are considered examples of behavioral impairment^9^. Other behavioral symptoms of dementia include sleep disorders^10^, which are common among older adults (e.g., insomnia, circadian rhythm disorder, Willis–Ekbom disease/restless legs syndrome [WED/RLS])^11^. WED/RLS is a condition that has been investigated in older adults with dementia^12^.

WED/RLS can occur in any phase of life. The prevalence increases in adulthood, with a slight drop among older adults^13,14^. WED/RLS is characterized by the need to move the lower limbs during rest, especially at night, as the movement provides a type of relief^15^. This condition is associated with a reduction in total sleep duration, poor sleep quality, and diminished quality of life^16,17^.

The difficulty individuals with dementia have in reliably describing their own clinical status hampers the WED/RLS diagnosis, which implies underdiagnosis or misdiagnosis^14,18^. The WED/RLS diagnosis is based on self-reports of the desire to move the legs, accompanied by a sensation of pain, tingling, a burning sensation, and itching^19^.

Although no lesions have been identified in specific regions of the central nervous system, WED/RLS constitutes a disorder of this system^20^. Aspects associated with WED/RLS have been reported in the literature^17^, such as genetic factors (e.g., a family history of WED/RLS)^21^, dysfunctional dopaminergic neurotransmission, and abnormalities in the central metabolism of iron. Other factors, such as chronic conditions (e.g., diabetes)^22^ and the use of medications (e.g., antidepressants)^23^, can precipitate or aggravate the syndrome.

Untreated WED/RLS in older adults with dementia can have significant consequences, such as nocturnal agitation, discomfort, pain, falls, and diminished quality of life^24^. Some studies have investigated the prevalence of WED/RLS in older adults with dementia^25,26^. In a systematic review, the prevalence ranged from 4 to 24%^12^. However, no studies on this issue have been conducted with Brazilian older adults. Therefore, the aims of the present study were to determine the prevalence of Willis-Ekbom disease/restless legs syndrome in a sample of older adults with dementia and investigate the association between ferritin levels and the WED/RLS diagnosis in older adults with dementia under care at a psychiatric hospital in Brazil.

## METHODS

### Ethical considerations

This study received approval from the Human Research Ethics Committee of the Bairral Institute of Psychiatry (certificate number: 4.933.251). Patients and/or legal guardians who expressed interest in participating in the study signed a statement of informed consent before completing the data collection instruments.

### Study design and participants

A cross-sectional study was conducted using a convenience sample of individuals attending follow-up appointments at a psychiatric clinic in the city of Itapira, state of São Paulo, Brazil. The sample included 70 older adults diagnosed with dementia. The inclusion criteria were patients of both genders, 60 years of age or older, with a diagnosis of MND, clinically stable (no change in medications in the previous 30 days), and absence of symptoms of influenza or other acute diseases. Furthermore, individuals in advanced stages of dementia, or those with severe vision and hearing impairments (without correction), were excluded from the study. The same criteria applied to caregivers, who were required not to be primary caregivers but rather secondary or tertiary caregivers.

### Measures

#### Restless legs syndrome

The Restless Legs Syndrome Rating Scale (RLSRS) was used to assess the severity and impact of WED/RLS symptoms. This scale is composed of 10 items with response options scored from 0 (none) to 4 (very severe). The total ranges from 0 to 40^27^.

#### Neuropsychiatric symptoms

The Neuropsychiatric Inventory (NPI) addresses 12 categories of neuropsychiatric symptoms: delusions, hallucinations, agitation, depression/dysphoria, anxiety, euphoria, apathy, disinhibition, irritability/lability, abnormal motor behavior without purpose, sleep disorders and nighttime behaviors, appetite, and eating disorders.^28^ The NPI is used to record the frequency (1 [absent] to 4 [very often]) and intensity (1 [mild] to 3 [severe]) of symptoms.

#### Cognition

The Mini-Mental State Examination (MMSE) is composed of eight questions distributed among seven categories: temporal orientation, spatial orientation, registration of three words, attention and calculation, recall of three words, language, and visuo-constructive capacity.^29^ The score ranges from 0 to 30 points. The MMSE is a cognitive screening scale with a good correlation with the evolution of dementia. Different cutoff points are used based on schooling level: 20 (illiterate), 25 (1–4 years of schooling), 26.5 (5–8 years), 28 (9–11 years), and 29 (more than 11 years of schooling).^29^

#### Sleep quality

The Pittsburgh Sleep Quality Index is a self-assessment questionnaire for the investigation of sleep and disorders in the previous month. The index has 19 items distributed among the following domains: subjective sleep quality, sleep latency, sleep disturbances, sleep duration, daytime dysfunction, habitual sleep efficiency, and use of sleeping medications. The response options are on a Likert scale ranging from 0 to 3 points. The total ranges from 0 to 20 points, with higher scores denoting poorer sleep quality. The Brazilian version of PSQI-BR has satisfactory sensitivity and reliability (Cronbach's alpha coefficient = 0.68)^30^.

#### Sleepiness

The Epworth Sleepiness Scale (ESS) is a self-administered questionnaire that furnishes a measure of an individual's general level of daytime sleepiness. The scale has eight items that address the subjective assessment of the likelihood of dozing in different situations. Each item is scored on a Likert scale ranging from 0 (no chance of dozing) to 3 (high chance of dozing). The total ranges from 0 to 24 points. A score of 10 points is the cutoff that differentiates normal individuals from those with sleep disorders, such as obstructive sleep apnea syndrome, narcolepsy, and idiopathic hypersomnia. The translated version validated for use in Brazil was used, which has high sensitivity and specificity (Cronbach's alpha coefficient=0.76)^31^.

### Procedures

Interviews were held between February and December 2022. A convenience sample was recruited from patients waiting for a medical appointment at the clinic of the Bairral Psychiatric Hospital, where the researchers presented the objectives of the study. Individuals who met the eligibility criteria and agreed to participate were taken to a reserved room at the hospital for data collection, which lasted a mean of 60 min. Prior to answering the data collection instruments, the family members authorized participation by signing a statement of informed consent.

The patients answered a questionnaire designed by the researchers addressing age, gender, marital status, schooling, and employment status. The diagnosis of MND was established based on the criteria of the 5th edition of the Diagnostic and Statistical Manual of Mental Disorders (DSM-5)^32^. Patients who had dementia diagnoses, according to the information available in the medical records of patients treated in the outpatient hospital where the study was conducted, were invited for WED/RLS evaluation. The diagnostic criteria of the International Restless Legs Syndrome Study Group were used for the WED/RLS determination (1 = desire to move the limbs; 2 = motor restlessness; 3 = worsening or presence of symptoms only at rest, and 4 = worsening of symptoms in the evening or at night) according to the most recent edition of the International Classification of Sleep Disorders-3^33^. None of the patients had a previous WED/RLS diagnosis. To perform the diagnosis of this condition (i.e., WED/RLS), psychiatrists were trained to perform this assessment. In addition to the RLSRS measure, these professionals made a clinical anamnesis to highlight other conditions (e.g., neuropathy, cramps, and others).

As the study was conducted during the COVID-19 pandemic, all protective measures were taken. Each assessment lasted a mean of 60 minutes with caregivers. All measures were completed by caregivers, except for MMSE, which was applied to patients (i.e., patients with dementia). Besides the data collection instruments described above (i.e., RLSRS, NPI, MMSE, PSQI-BR, and ESS-BR) and the sociodemographic questionnaire, blood exams were performed for the determination of creatinine, ferritin, red blood cells, hemoglobin, and hematocrit.

### Statistical analysis

The data were analyzed with the aid of the SPSS program (25.0). Descriptive statistics (percentage, mean, and standard deviation) were performed for the characterization of the sample with regard to sociodemographic and clinical data. The Kolmogorov-Smirnov test identified that most of the variables were parametric. Skewness and kurtosis values equal to or less than 2.58 (positive or negative)^34^ were considered for variables with a p-value <0.05 in the normality test. The chi-square test and student's t-test were used for the comparison of categorical and continuous variables, respectively. The significance level for all analyses was set at 5% (p≤0.05).

## RESULTS

Table 1 displays the characteristics of the sample, which was composed of 70 older adults with dementia. Women predominated (55.7%), and mean age was 77.80±9.36 years. No significant differences were found between the groups with and without WED/RLS in relation to the sociodemographic variables. The prevalence of WED/RLS was 15.7%.

**Table 1:** Sociodemographic characteristics of patients with and without Willis-Ekbom disease/restless legs syndrome.

Variables (n (%) or mean±SD)		Total (n=70)	With WED/RLS (n=11)	Without WED/RLS (n=59)	χ^2^/U	p-value
Age		77.80 (±9.36)	76.11 (±7.82)	78.10 (±9.65)	0.621	0.537
Gender						
	Female	39 (55.7)	5 (45.5)	34 (57.6)	0.557	0.456
	Male	31 (44.3)	6 (54.5)	25 (42.4)		
Marital status						
	Married	22 (31.4)	3 (27.3)	19 (32.2)	0.985	0.805
	Single	12 (17.1)	1 (9.1)	11 (18.6)		
	Widowed	30 (42.9)	6 (54.5)	24 (40.7)		
	Divorced	6 (8.6)	1 (9.1)	5 (8.5)		
	Schooling (year)	4.33 (±3.71)	5.82 (±3.92)	4.05 (±3.63)	-1.459	0.149
Ethnicity						
	White	46 (65.7)	7 (63.6)	39 (66.1)	-	-
	Black	6 (8.6)	2 (18.2)	4 (6.8)		
	Brown	16 (22.9)	1 (9.1)	15 (25.4)		
	Asian	2 (2.9)	1 (9.1)	1 (1.7)		
Employment status						
	Retired	63 (90.0)	10 (90.9)	53 (89.8)	-	-
	Unemployed	5 (7.1)	-	5 (8.5)		
	Social security benefits	2 (2.9)	1 (9.1)	1 (1.7)		

Abbreviations: SD, standard deviation; WED/RLS, Willis-Ekbom disease/restless legs syndrome.

Table 2 displays the clinical characteristics of the patients with and without WED/RLS. A large portion of the sample (42.9%) had a diagnosis of mixed dementia (i.e., Alzheimer's + vascular dementia). Patients with WED/RLS had greater frequencies of neuropsychiatric symptoms (p=0.026) and poor sleep quality (p=0.001) as well as a higher BMI (p=0.029) and lower ferritin levels (p=0.002) compared to those without WED/RLS. Patients with WED/RLS also had significantly worse scores (p=0.001) on all domains of the PSQI (subjective sleep quality, sleep latency, sleep disturbances, sleep duration (i.e., frequencies between 6 and 7 h), daytime dysfunction, habitual sleep efficiency, and use of sleeping medications).

**Table 2:** Clinical characteristics of patients with and without restless legs syndrome.

Variables (n (%) or mean±SD)		Total (n=70)	With WED/RLS (n=11)	Without WED/RLS (n=59)	χ^2^/U	p-value
Cognition – MMSE		13.13 (±5.95)	14.27 (±8.90)	12.92 (±5.30)	11.362	0.364
Frequency – NPI		8.97 (±5.28)	12.55 (±7.76)	8.31 (±5.21)	-2.282	0.026*
Severity – NPI		13.72 (±10.42)	20.72 (±12.58)	12.42 (±9.35)	12.232	0.059
Sleepiness – ESS†		4.13 (±4.36)	6.09 (±3.91)	3.75 (±4.38)	-1.645	0.105
Sleep quality – PSQI		4.22 (±4.47)	11.63 (±4.00)	2.84 (±2.94)	-8.563	0.001*
Subjective sleep quality		0.81 (±1.01)	2.00 (±0.89)	0.59 (±0.87)	4.807	0.001*
Sleep latency		0.73 (±0.96)	2.00 (±1.00)	0.50 (±0.75)	4.726	0.001*
Sleep duration		0.51 (±0.81)	1.45 (±1.03)	0.33 (±0.63)	4.807	0.001*
Sleep efficiency		0.42 (±0.75)	1.36 (±0.92)	0.25 (±0.57)	5.290	0.001*
Sleep disturbances		0.32 (±0.73)	1.18 (±0.98)	0.16 (±0.56)	4.809	0.001*
Use of sleeping medications		0.95 (±1.13)	2.27 (±1.19)	0.71 (±0.94)	12.471	0.001*
Daytime dysfunction		0.44 (±0.84)	1.36 (±1.20)	0.27 (±0.63)	4.438	0.001*
BMI^†^		25.76 (±4.36)	28.37 (±4.21)	25.26 (±4.24)	-2.226	0.029*
Ferritin‡		128.32 (±115.58)	59.15 (±19.08)	136.46 (±119.57)	3.418	0.002*
Hemogram						
	Hemoglobin§	13.52 (±1.71)	14.65 (±3.03)	13.38 (±1.33)	0.234	0.234
	Hematocrit//	40.72 (±5.06)	43.81(±9.11)	40.1 (±3.81)	0.268	0.268
Comorbidities		4.74 (±2.24)	5.64 (±2.06)	4.58 (±2.25)	-1.542	0.152
Number of medications taken		6.51 (±3.58)	6.09 (±3.08)	6.59 (±3.68)	0.424	0.673
Use of antidepressants						
	Yes	45 (64.3)	8 (72.7)	37 (62.7)	0.394	0.394
	No	25 (35.7)	3 (27.3)	22 (37.3)		

Abbreviations: SD, standard deviation; WED/RLS, Willis-Ekbom disease/restless legs syndrome; MMSE, Mini-Mental State Examination; NPI, Neuropsychiatric Inventory; ESS, Epworth Sleepiness Scale; PSQI, Pittsburgh Sleep Quality Index; BMI, body mass index.

* Notes: p<0.05;

† sample of 68 patients for this variable;

‡ sample of 38 patients for this variable;

§ sample of 62 patients for this variable;

// sample of 58 patients for this variable.

## DISCUSSION

This study investigated the frequency of WED/RLS and ferritin deficiency in older adults with dementia of different etiologies. The prevalence of WED/RLS was 15.7%, and individuals with this diagnosis had lower ferritin levels. The results also showed that individuals with a diagnosis of the syndrome had greater frequencies of neuropsychiatric symptoms and poor sleep quality.

The prevalence of WED/RLS in this study (15.7%) was higher than that reported in a previous Brazilian study conducted with a population of older adults (6.4%)^13^. A review study reported 4–24% rates^12^, although the prevalence was higher than 20% in previous studies involving older adults with a dementia diagnosis^18^. A possible explanation for the divergent results is the method used for the syndrome diagnosis. The present study used the criteria of the International Classification of Sleep Disorders-3^33^, which are considered the "gold standard," whereas the studies with higher rates^18^ involved non-standard assessments, which may explain the divergence.

The literature reports that the world prevalence of WED/RLS is 3%^34^, whereas the prevalence in Brazil is more than double this rate, reaching 6.4%^13^. The higher prevalence found in the present study may be justified by the sample, as the literature reports a higher frequency of the syndrome in older adults^35^. A study conducted with 1,803 individuals of both genders investigated the prevalence of restless legs syndrome in different age groups (18–29 years, 30–79 years, and 80 years or older) and found an increasing rate of 3, 10, and 19%, respectively^36^. The prevalence in the present study falls within the range of 10–19% and the mean age of the participants was 77.80 years. Another factor that may explain the prevalence is the greater presence of women in the sample. Although no significant difference in gender was found between the groups, previous studies^13^ demonstrated that women are more likely to be diagnosed with the syndrome.

The biological relationship between these variables is not well-established in the literature. However, some factors may contribute, such as the decrease in white and gray substances that occurs in patients with WED/RLS and is observed in patients with dementia^37,38^. Moreover, the presence of cardiovascular diseases is highlighted, since studies have shown that individuals with this syndrome present unfavorable cardiovascular conditions^39,40^. As we know, these unfavorable conditions are a risk factor for dementia^41^.

WED/RLS can exert negative impacts on affected individuals and can be related to poor sleep quality^17^. Indeed, the older adults with dementia and WED/RLS in the present study had poor sleep quality. In a review study, Richards et al.^17^ found that older adults with dementia and a diagnosis of WED/RLS may have sleep disorders, which, in turn, implies poor sleep quality. This finding is in agreement with data described in studies conducted with healthy older adults^42^. A possible explanation is the presence of a chronic condition in the patients of the present study (i.e., dementia), as previous studies identified an association between chronic conditions (e.g., dementia) and sleep quality^43^.

Another explanation for poor sleep quality in patients with WED/RLS is the high BMI (28.37 kg/m^2^), which is considered indicative of excess weight^44^. Some studies have investigated the association between sleep quality and BMI^45^. Wallen et al.^46^ found that individuals with a high BMI have poor sleep quality. Besides poor sleep quality caused by BMI, studies have also found that this factor is related to WED/RLS^46^. The increase in weight and, possibly, adipose tissue can increase levels of inflammatory mediators^47^, which, in turn, may be associated with worse symptoms of WED/RLS and can lead to a reduction in serum iron levels, as identified in a study involving individuals with obesity^48^.

Greater frequencies of neuropsychiatric symptoms were found in the group with WED/RLS. The presence of psychiatric symptoms is common among older adults with dementia^9^. A recent systematic review found psychiatric symptoms in dementia of different etiologies^6^, and psychiatric symptoms are common among individuals with WED/RLS. Moreover, a review found that low ferritin levels are associated with psychiatric manifestations^49^.

In the present study, older adults with dementia and a diagnosis of WED/RLS had ferritin deficiency. Ferritin levels often increase in healthy older adults^50^. However, serum ferritin levels diminish in those with dementia, especially in the presence of neurodegenerative diseases (e.g., Alzheimer's disease)^51^. Moreover, low ferritin levels are associated with an increase in WED/RLS^52^. Indeed, being 60 years of age or older, having a chronic condition (i.e., dementia), and low ferritin levels contribute to the presence of WED/RLS.

One study found that individuals with WED/RLS have ferritin levels ≤50 mcg/L^53^, whereas higher levels were found in the present study (i.e., >50 mcg/L). However, this aspect is not well-established, as divergent findings are described in the literature, with some studies reporting that the ferritin level is associated with WED/RLS in older adults with dementia^54^ and others reporting no association^55^. Although we found this association in the present investigation, further studies are needed, especially those with a longitudinal design, to determine the relationship between the variables.

This study has limitations that should be considered, such as the use of a convenience sample, the small sample size, the cross-sectional design, which impedes the inference of causality, the lack of information on some clinical variables (i.e., complementary laboratory exams), which resulted in the comparison of an even smaller number of individuals, and the non-use of polysomnography, which impeded us from assessing the presence and impact of periodic limb movements. Moreover, we could not rule out the presence of sleep apnea as a confounding variable for our findings.

In conclusion, in the present study, the prevalence of Willis–Ekbom disease/restless legs syndrome was 15.7% in a sample of older adults with dementia. WED/RLS was associated with poor sleep quality, a greater frequency of psychiatric symptoms, a higher BMI, and lower ferritin levels. These findings are unprecedented and underscore the importance of the identification of the presence and consequences of WED/RLS in older adults with dementia. This is the first Brazilian study involving older adults with different etiologies of dementia and a diagnosis of WED/RLS.
